# The Ubiquitin-Specific Protease Family: Master Regulators of Renal Fibrosis Pathogenesis and Therapeutic Targets

**DOI:** 10.3390/ijms27052318

**Published:** 2026-03-01

**Authors:** Yinhang Wang, Dadui Ren, Wenjun Zhao, Yongmei Zhang, Xuemei Zhang

**Affiliations:** 1 Joint Center for Translational Medicine, Shanghai Fifth People’s Hospital, Fudan University, & School of Pharmacy, East China Normal University, Shanghai 200241, China; 2 Department of Neurology, Xuanwu Hospital Capital Medical University, National Center for Neurological Disorders, Beijing 100053, China; zwj90303@126.com; 3 Institute of Biomedical Research, Henan Academy of Sciences, Zhengzhou 450046, China; rendd@hnas.ac.cn; 4 Innovation Center for AI and Drug Discovery, School of Pharmacy, East China Normal University, Shanghai 200062, China; ymzhang@pharm.ecnu.edu.cn

**Keywords:** ubiquitin-specific protease, renal fibrosis, deubiquitination

## Abstract

Ubiquitin-specific proteases (USPs) constitute the largest and most diverse family of deubiquitinating enzymes (DUBs), playing a pivotal role in maintaining protein homeostasis through reversible post-translational modifications (PTMs). Renal fibrosis represents the final common pathway of various chronic kidney diseases (CKDs), ultimately leading to irreversible nephron loss and end-stage renal disease (ESRD). With CKD affecting over 10% of the global adult population, fibrosis imposes a substantial clinical and economic burden. Despite this, effective antifibrotic therapies remain clinically elusive. Emerging evidence highlights the critical involvement of USPs in the pathogenesis of renal fibrosis through the potentiation of pro-fibrotic signaling pathways, inflammation, oxidative stress, cell cycle arrest and cellular senescence, as well as some other pathways. This review comprehensively summarizes the current understanding of USPs in renal fibrosis, detailing their structural characteristics, molecular mechanisms, and specific regulatory roles. Furthermore, we discuss recent advances in developing small-molecule USP inhibitors, providing novel insights into targeting the ubiquitin–proteasome system as a promising therapeutic strategy for combating renal fibrosis.

## 1. Introduction

Renal fibrosis, the pathological hallmark and final common pathway of chronic kidney disease (CKD), represents a staggering global health burden, affecting 788 million adults worldwide as of 2023 [1]. Unlike pulmonary or hepatic fibrosis, where diagnostic imaging and biopsy protocols are well-established for monitoring progression, renal fibrosis poses unique challenges due to the kidney’s complex cellular heterogeneity and the “silent” nature of its progression. Consequently, clinical interventions are severely limited; while drugs like pirfenidone have been approved for pulmonary fibrosis, there are currently no food and drug administration (FDA)-approved therapies capable of effectively reversing renal fibrosis without significant off-target toxicity [2,3]. This therapeutic impasse underscores the urgent need to decipher the molecular drivers of fibrogenesis. Recent mechanistic studies have identified the ubiquitin–proteasome ubiquitin-proteasome system as a critical regulator of renal homeostasis. Specifically, Ubiquitin-Specific Proteases (USPs)—the largest subfamily of deubiquitinating enzymes (DUBs)—act as “molecular surgeons” within the renal tubular and interstitial compartments. By precisely cleaving ubiquitin chains from substrate proteins, USPs rescue key signaling mediators (e.g., TGF-βreceptors, transduction factors) from proteasomal degradation or modulate their non-proteolytic functions [4], and then regulate the progression of renal fibrosis in different pathways. In the fibrosis process, the research on the regulatory roles of USPs in different types of renal cells across various pathways is not well-developed, and the lack of targeted research on selective USP inhibitors has become a key knowledge gap in this field. To address these gaps, the scope of this review is to systematically delineate the precise mechanisms by which the USP family regulates profibrotic pathways, providing a rationale for targeting individual USPs to develop precision anti-fibrotic therapies.

## 2. Literature Search Strategy

To ensure a comprehensive and rigorous narrative review, an extensive literature search was conducted across electronic databases, primarily utilizing PubMed and Google Scholar. Although no strict publication timeframe restrictions were imposed in order to capture the foundational discoveries of the ubiquitin–proteasome system, the study selection heavily prioritized recent, high-impact, and cutting-edge original research and reviews. The search strategy utilized a wide array of Medical Subject Headings (MeSH) terms and free-text keywords, employing Boolean operators (AND, OR) to construct both broad and highly specific queries. The standardized search strings included combinations of one, two, or three core concepts, such as: (“Ubiquitin-Specific Protease*” OR “USP*” OR “deubiquitinating enzyme*” OR “DUB*” OR “ ubiquitin-proteasome system*” OR “ UPS*”) AND (“renal fibrosis” OR “kidney fibrosis” OR “chronic kidney disease” OR “CKD”) AND (“pathogenesis” OR “therapeutic target*” OR “inhibitor*” OR “medicine”). Searches were systematically iteratively refined to maximize search sensitivity and specificity. The inclusion criteria strictly focused on peer-reviewed English-language articles, high-quality mechanistic in vitro and in vivo studies, and recent pharmacological advancements regarding USP-targeted drug development. Articles were critically screened by the authors and selected based on their direct relevance to the USP family’s specific pathophysiological mechanisms in renal fibrogenesis, while non-English publications and unpublished preprints were excluded.

## 3. Ubiquitin-Specific Proteases

### 3.1. Origins and Evolution of USP

**Figure 1:**
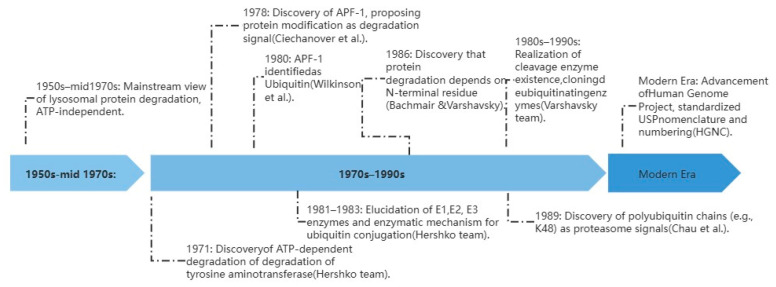
The ubiquitin-specific proteases (USPs) timeline of research spans from the mid-1950s to the present. From the discovery of ubiquitin to the standardized naming of USPs.

The identification of the USP family represents a milestone in understanding intracellular protein quality control, closely mirroring the paradigm shift from lysosomal degradation to the ubiquitin–proteasome system. While early research established the ATP-dependent ubiquitin-conjugation cascade, the necessity of a reverse mechanism—deubiquitination—was identified to maintain the intracellular free ubiquitin pool. Ultimately, in the process of studying ubiquitin gene expression, the first deubiquitinating enzyme genes (UBP1-3) were cloned. As research shifted to mammals, the nomenclature was standardized by the Human Genome Nomenclature Committee (HGNC) to USP (Ubiquitin-Specific Protease), reflecting its highly conserved papain-like cysteine protease core and its broader function of removing ubiquitin from target substrates [5,6]. The USP family, the largest of the seven distinct subfamilies of DUBs, contains 56 members in humans and has a profound impact on a wide range of biological processes [7,8,9]. Crucially, emerging evidence highlights the USP family as a master regulator in the pathogenesis of renal fibrosis, directly modulating fibrogenic signaling, cellular senescence, and extracellular matrix accumulation. And the distribution of USP family members in human tissues and cells exhibits a high degree of functional correlation (Figure 1).

### 3.2. Structure and Function of USPs

#### 3.2.1. Classic USP Catalytic Domains

The structural landscape of the USP family is defined by a highly conserved catalytic core that adopts a characteristic “extended right-hand” topology. This architecture comprises three subdomains: the Finger, which facilitates ubiquitin capture; the Palm, housing the catalytic center; and the Thumb, which coordinates the ubiquitin C-terminus to create a deep, solvent-accessible cleft for isopeptide bond hydrolysis [10,11,12]. As cysteine proteases, USPs utilize a classical catalytic triad—typically comprising Cysteine, Histidine, and Aspartate/Asparagine—to execute nucleophilic attack, forming a tetrahedral intermediate that resolves into an acyl–enzyme intermediate before final hydrolysis [13]. While the catalytic triad is structurally conserved, its spatial arrangement often exhibits remarkable plasticity, existing in “unproductive” conformations in apo-enzymes like USP7 and USP14 to prevent non-specific reactivity. For instance, the active site of free USP7 is misaligned with the catalytic Cys223 and His464, which are too far apart for catalysis, requiring a substrate-induced “switch” loop movement and C-terminal tail engagement to lock the triad into a competent state [13,14,15,16]. This conformational plasticity is further governed by steric gating mechanisms, such as the surface loops BL1 and BL2 in USP14 that physically block the catalytic groove until proteasome binding or AKT-mediated phosphorylation at Ser432 induces their displacement, thereby granting ubiquitin access [17,18,19].

#### 3.2.2. Structural Determinants of Substrate-Specificity

While all USPs recognize ubiquitin, they exhibit exquisite specificity for distinct ubiquitin chain linkages (e.g., K48, K63, K11, linear chains) and substrate contexts. This specificity is determined by the fine topology of the enzyme surface. Most USP domains have at least two ubiquitin-binding sites, coordinating both proximal and distal ubiquitin molecules. These sites can cleave the isopeptide bond between the proximal ubiquitin and the protein substrate, as well as the next ubiquitin molecule linked to the distal ubiquitin [12]. Structural complexity is further enhanced by modular insertions and extensions, and many USPs contain auxiliary ubiquitin-binding domains (UBDs), such as the Zinc-finger ubiquitin binding protein (ZnF-UBP) domains acting as “chain catchers” or allosteric activation sensors [20,21]. These features enable USPs to precisely cleave the isopeptide bond between the proximal ubiquitin and the substrate, or within polyubiquitin chains.

#### 3.2.3. Regulatory Domains and Modular Assembly

The USP catalytic core rarely functions in isolation; it is frequently punctuated by large insertion sequences or flanked by N/C-terminal extensions. These regions form independent folded structural domains, which are evolutionary hotspots and the center of functional regulation. Sequence alignment divides the USP catalytic core into six conserved “boxes” (Box 1–6). Large-scale sequence insertions frequently occur between these conserved boxes, especially between Box 3 and 4, and between Box 4 and 5. A prominent example is the DUSP-UBL unit found in the USP4/11/15 subfamily, where an N-terminal USP-specific protease domain (DUSP) and a ubiquitin-like domain (UBL) are tightly stacked via a conserved VEVY (Val-Glu-Val-Tyr) motif [12,22,23,24]. Furthermore, UBL domains in USP typically mimic the folding of ubiquitin to “deceive” or recruit binding partners or even exert intramolecular auto-regulation. For example, the C-terminal extension region of USP7 forms a rigid substrate recognition platform through the tandem arrangement of up to 5 UBL domains, while the peptide at the extreme C-terminus folds back and binds to the catalytic domain, locking it in an active conformation [15,23]. Unlike the structural zinc ribbon in the finger domain, the ZnF-UBP domain is an independent fold with a deep ubiquitin-binding pocket and is found in USP5, USP13, and USP20. For instance, the ZnF-UBP domain at the N terminus of USP20 forms a spherical fold. Although its sequence is highly similar to that of USP5, structural analysis shows that the ZnF-UBP of USP20 exhibits low affinity for ubiquitin. This suggests that in USP20, the domain has evolved to recognize specific substrate conformations or other protein ligands, rather than serving as a generic ubiquitin binder [20].

#### 3.2.4. Allosteric Regulation: Structural Mechanisms of Activation and Inhibition

Many USPs possess low intrinsic activity and rely on “on-demand activation” via specific binding partners or post-translational modifications (PTMs) to become activated. This “on-demand activation” strategy is crucial for preventing dysregulation of intracellular ubiquitin signaling. Recent cryo-EM studies on USPs have captured its dynamic transition upon binding to full-length DNA methyltransferase 1 (DNMT1) (DNA methyltransferase 1), confirming previous static studies and conjectures. It was found that USP7 was still inactive when only the RFTS domain of DNMT1 was bound. However, when combined with full-length DNMT1 and ubiquitinated histone H3, the substrate DNMT1 itself acts as an allosteric effector, inducing a drastic conformational transition from open (inactive) to closed (active) USP7. Then, based on structural design, small molecule activators (such as MS-8) were developed, which can mimic the binding of endogenous C-terminal peptides to allosteric pockets and forcibly lock the enzyme to the active conformation [23,25]. Other allosteric modulations include, for example, USP14 activation dependent on the displacement of surface blocking rings (BL1 and BL2), which is achieved through both proteasome-mediated and phosphorylation-mediated pathways [18,19,26]. The WW-like domain of USP8 refracts back and covers the catalytic center, forming a self-inhibiting conformation. The 14-3-3 protein stabilizes this self-inhibitory conformation like a lock by binding to the phosphorylated residues (e.g., Ser718), thus preventing aberrant activation [27,28].

#### 3.2.5. Other Structural–Functional Regulation Modalities

Beyond intramolecular regulation, several USPs require obligate cofactors for stability and activity. USPs with WD40-repeat (WDR) partners, such as USP1 (with UAF1), USP12, and USP46, exemplify this paradigm, where the cofactor is essential for catalytic competence [29,30,31,32]. Similarly, USP22 (yeast Ubp8) is catalytically inert in isolation and functions only within the SAGA deubiquitination module (DUBm), composing ATXN7 (Sgf73), ATXN7L3 (Sgf11), and ENY2 (Sus1). This assembly precisely positions USP22 to deubiquitinate histone H2B-K120, linking structural interlocking to epigenetic regulation [33,34,35,36]. Additionally, oligomerization dictates function in the USP25/28 pair, where differences in autoinhibitory motifs lead to distinct oligomeric states and functional outcomes despite high catalytic sequence identity [37,38].

## 4. Pathophysiological Mechanisms of USPs in Renal Fibrosis

### 4.1. Pro-Fibrotic Signaling Pathways

#### 4.1.1. TGF-β/SMAD Signaling

Transforming Growth Factor-beta 1 (TGF-β1) stands as the preeminent pro-fibrotic cytokine driving renal fibrosis among the three TGF-β isoforms in mammals [39,40]. TGF-β1 is secreted into the extracellular matrix (ECM) as a latent complex, composed of mature TGF-β dimers, latent-related peptide (LAP), and latent TGF-β-binding protein (LTBP). Upon injury, ROS, plasmin or integrins (such as αvβ6) induce conformational changes, releasing active TGF-β1 [41,42]. Active TGF-β1 binds to the extracellular domain of the type II receptor (TβRII) on the cell membrane. TβRII recruits and phosphorylates the glycine-serine enriched domain (GS domain) of the type I receptor (TβRI/ALK5), thereby activating the kinase activity of TβRI [43]. Following TβRI activation, it specifically phosphorylates downstream receptor-regulated SMADs (R-SMADs), primarily SMAD2 and SMAD3, regulating the balance between pro-fibrotic and renal protection. Phosphorylated R-SMAD (p-SMAD2/3) then forms a heterotrimeric complex with universal SMAD (Co-SMAD, i.e., SMAD4), which translocates into the nucleus [40,44,45]. Within the nucleus, the SMAD complex binds to coactivators or corepressors, regulating target gene transcription [46,47]. Furthermore, as an important negative feedback regulatory mechanism, TGF-β signaling induces SMAD7 expression, which in turn inhibits the signal through various mechanisms [48,49,50].

##### Deubiquitination Regulation of the TGF-β Receptors

**Figure 2:**
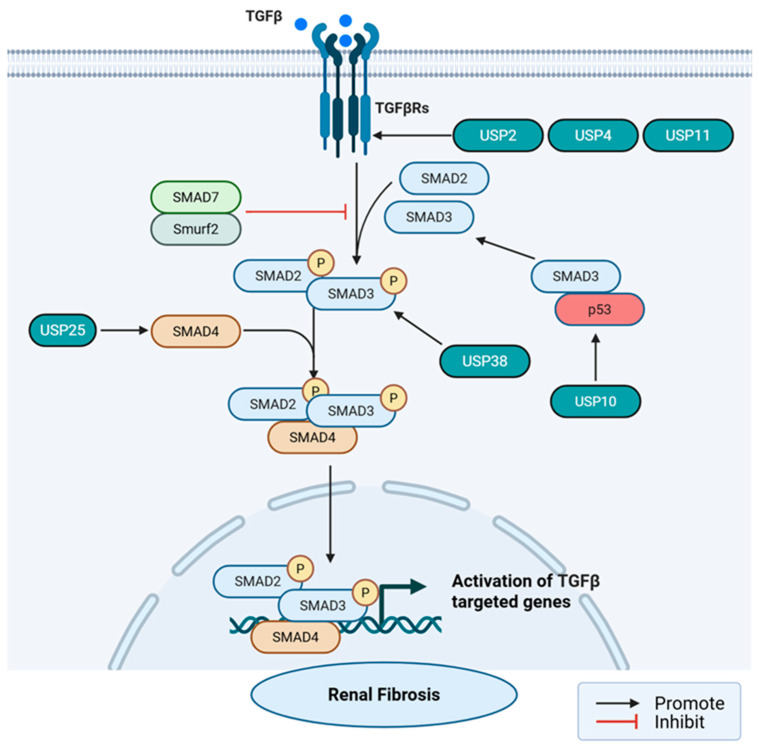
USPs regulate renal fibrosis through TGF-β/SMAD signaling. USP2, USP4, and USP11 stabilize TβRI/TβRII, promoting fibrosis. USP10 promotes fibrosis expression by ubiquitinating p53, forming a complex with SMAD3. USP25 stabilizes SMAD4, promoting fibrosis. USP38 promotes the phosphorylation and nuclear translocation of SMADSMAD2/3 through stable STRAP.

The regulatory mechanisms of USP4, USP11, and USP2 on the TGF-β receptors have been clearly validated in kidney fibrosis models (such as unilateral ureteral obstruction (UUO), folic acid nephropathy, diabetic nephropathy) or kidney cells. Meanwhile, the mechanisms of USP15 and USP8 regulating the TGF-β receptors are primarily elucidated in tumors or skin diseases, but similar mechanisms may exist in kidney fibrosis or require further validation. USP4 is the first USP member discovered to be able to directly reverse TGF-β receptor degradation [51]. When TGF-β signaling is initially activated, it induces the activation of the PI3K/AKT pathway. AKT directly phosphorylates USP4, promoting the rapid translocation of USP4 from the nucleus and cytoplasm to the cell membrane. USP4 binds directly to TβRI (ALK5) at the cell membrane. Under normal conditions, SMAD7 recruits the E3 ligase *Smurf2* to perform polyubiquitination modification on TβRI, leading to the receptor being sent to lysosomes or proteasomes for degradation. USP4 extends the half-life of TβRI on the membrane significantly by specifically removing the K48 ubiquitin chain on TβRI and spatially blocking the recognition of the receptor by the SMAD7–*Smurf2* complex [43,52]. Similarly supported by strong in vivo data, USP11 deubiquitinates TβRI to enhance the TGF-β signaling pathway [53]. In addition, USP11 removes K48 ubiquitin chains from TβRII through its deubiquitinating activity, preventing its entry into the lysosomal or proteasomal degradation pathway. It stabilizes TβRII and increases SMAD2/3 expression, inducing fibronectin (FN) and α-smooth muscle actin (α-SMA) [54]. Furthermore, recent high-quality evidence highlights a novel, indirect regulatory axis for USP2. In addition, the latest research found that USP2 does not directly remove ubiquitin from the receptor but removeubiquitin from the endoplasmic reticulum protein thioredoxin domain containing 5 (TXNDC5). The endoplasmic reticulum protein TXNDC5 acts as a molecular chaperone and is essential for the folding and stability of TβRI. USP2-deubiquitinated TXNDC5 maintains levels of TXNDC5, thereby stabilizing TβRI through TXNDC5 and promoting TGF-β signaling [55]. Similarly, USP15 enhances TGF-β signaling by deubiquitinizing TβRI [56]. USP15 forms a complex with *Smurf2* and SMAD7 and is recruited by SMAD7 to TβRI for deubiquitination and stabilization. In the presence of weak TGF-β signals, USP15 binds to the SMAD7–*Smurf2*2–TβRI complex and promotes TGF-β signaling, whereas upon exposure to strong TGF-β signals, USP15 dissociates from the SMAD7–*Smurf2*– TβRI complex and blocks TGF-β signaling, forming a delicate balance [57]. This biphasic nature indicates that targeting USP15 in chronic, high-TGF-β environments like progressive renal fibrosis may yield unpredictable or contradictory outcomes, requiring cautious in vivo evaluation before being considered a viable therapeutic target. USP8 primarily functions at the endosome level. It regulates receptor sorting in endosomes by deubiquitinating TβRII, preventing its entry into lysosomes for degradation and promoting its recycling back to the cell membrane, thereby enhancing the cell’s sensitivity to TGF-β [58] (Figure 2).

##### Stability and Nuclear Translocation Regulation of SMAD Media

In the process from the membrane to nuclear entry, USP10, USP2a, USP38, and USP25, which have already played a role in renal fibrosis or nephropathy, and USP7, USP15, USP10, etc., which have been confirmed to be involved in SMAD-mediated regulation in other organs and diseases, are significantly involved, and there are also some cross-actions of signaling pathways involved. In CKD studies, it was found that USP10 directly binds to p53 and removes its K48-linked polyubiquitin chains, thereby stabilizing p53 against degradation, and subsequently forms a transcriptional complex with phosphorylated SMAD3. The p53-SMAD3 complex binds to the promoters of fibrotic genes, strongly driving their expression [59,60,61]. In the CKD model, research has found that USP38 stabilizes Serine-Threonine Kinase Receptor-Associated Protein (STRAP) through ubiquitinase inhibition and promotes phosphorylation and nuclear translocation of SMAD2/3, thereby enhancing the TGF-β/SMAD signaling pathway [62]. TGF-β stimulation typically induces K63-linked polyubiquitination of SMAD4, which is crucial for the formation of the SMAD2/3-SMAD4 complex and its nuclear entry. Studies have found that in renal tubular-related diseases (HRD), USP25 specifically recognizes and removes the K63 ubiquitin chain on SMAD4 [63] (Figure 2).

In pulmonary fibrosis, studies have found that USP7 promotes TGF-β signaling by deubiquitinizing SMAD2/SMAD3 [64]. In the model of heart failure with preserved ejection fraction, USP7 was found to directly bind SMAD3 via the UBL domain, promoting SMAD3 stability by removing K63 ubiquitin chains, thereby preventing SMAD3 degradation by the proteasome. Here, K63 cleavage is considered to block degradation, suggesting the complexity of ubiquitin chain function in specific contexts [65]. Furthermore, mechanisms identified in oncology or developmental biology highlight precise structural regulation of SMADs, but their translational relevance to kidney fibrosis remains speculative and requires stronger in vivo corroboration. Research has found that monoubiquitination of SMAD3 inhibits its ability to bind to DNA. USP15 can remove this monoubiquitination modification, restoring SMAD3’s DNA-binding activity and thereby enhancing TGF-β transcriptional output. This mechanism is separate from the regulation of TβRI stability, demonstrating USP15’s multi-point control over the pathway [66]. In developmental biology and tumor cells, the E3 ubiquitin ligase TIF1γ (Ectodermin) adds a monoubiquitin molecule to lysine 519 (K519) of SMAD4. This modification does not lead to SMAD4 degradation, but its large size creates steric hindrance, physically blocking the binding of the SMAD4 MH2 domain to phosphorylated SMAD2/3. USP9X specifically removes this monoubiquitin modification [67]. Finally, the molecular targets of a single USP can be highly tissue-dependent, further complicating systemic therapeutic targeting. In liver cancer research, USP10 directly deubiquitinates and stabilizes SMAD4, promoting TGF-β signal maintenance and tumor metastasis, which differs from the p53-dependent SMAD regulation in kidney disease models, reflecting the diversity of regulation [68]. This differs from the p53-dependent (and SMAD4-independent) observed in kidney disease models, and these differences emphasize that the mechanism paradigm cannot be simply extrapolated across tissues, requiring experimental determination of the exact molecular substrates of these USPs in the fibrotic kidney microenvironment.

##### Regulation of Negative Feedback Loop

Exploiting the endogenous negative feedback loop of TGF-β signaling by stabilizing the inhibitory protein SMAD7 represents a theoretically attractive anti-fibrotic strategy; however, robust in vivo validation of USPs mediating this process in the kidney is currently lacking. As part of a negative feedback loop, the inhibitory SMAD protein SMAD7 is regulated by TGF-β and modulates TGF-β signaling gain through multiple mechanisms. SMAD7 not only recruits E3 ligases for receptor degradation, but it itself is also a substrate for the E3 ligases Arkadia and *Smurf*, undergoing self-ubiquitination degradation. Currently, the evidence linking specific USPs to SMAD7 stabilization is primarily extrapolated from non-renal tissues, placing them at a lower priority for immediate therapeutic translation in chronic kidney disease. In studies of insulin resistance-associated fibrosis and astrocytoma, USP26 is localized in the nucleus and can remove the K48-linked polyubiquitin chains on SMAD7, thereby stabilizing the SMAD7 protein. TGF-β stimulation can transiently induce USP26 expression, forming a negative feedback enhancement loop [69,70]. USP2 interacts with SMAD7, preventing SMAD7 ubiquitination in glioblastoma [71].

#### 4.1.2. Wnt/β-Catenin Signaling

**Figure 3:**
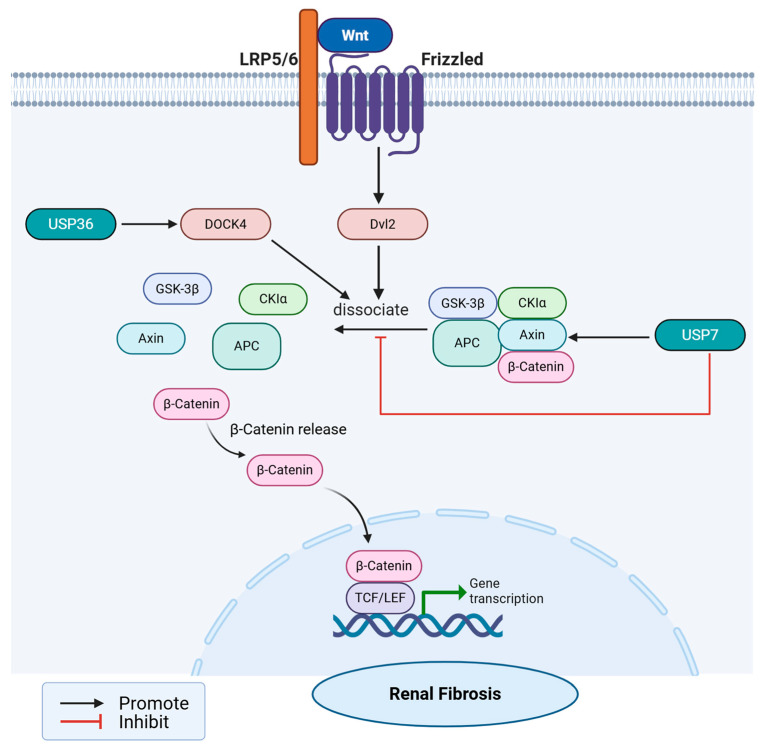
USPs regulate renal fibrosis through Wnt/β-catenin signaling. USPs regulate renal fibrosis through Wnt/β-catenin signaling. USP36 promotes Wnt/β-catenin signaling by stabilizing *DOCK4*. USP7 inhibits Wnt/β-catenin signaling by stabilizing Axin.

Wnt ligands bind to Frizzled receptors and LRP5/6 co-receptors on the cell membrane, inhibiting the intracellular β-catenin degradation complex, leading to the accumulation and nuclear entry of β-catenin, thereby regulating the expression of target genes such as Snail1, MMP7, and PAI-1, and directly promoting epithelial-to-mesenchymal transition (EMT) and fibrosis processes. At the same time, this pathway interacts with inflammatory pathways such as NF-κB [72,73,74]. As the largest subfamily of DUBs, USPs precisely regulate the stability of key components of the Wnt pathway by removing ubiquitin chains from substrate proteins, thereby controlling the progression of fibrosis. The roles of USP36 in the Wnt/β-catenin signaling pathway have been confirmed in kidney fibrosis or kidney disease models. Cytokinesis 4 (*DOCK4*) DOCK4 is a guanine nucleotide exchange factor (GEF) known to activate Rac1 and promote Wnt/β-catenin signaling. USP36 removes ubiquitin chains from *DOCK4*, preventing its degradation, leading to increased *DOCK4* protein levels. Stable *DOCK4* promotes the stability and nuclear translocation of Wnt/β-catenin, thereby activating the transcription of downstream Wnt target genes. This results in EMT in renal tubular cells, characterized by downregulation of E-cadherin and upregulation of α-SMA and Vimentin, thereby exacerbating renal fibrosis [75]. Conversely acting as a potential endogenous inhibitor, USP7 stabilizes Axin through deubiquitination, thereby enhancing its function in destabilizing the β-catenin-destruction complex, ultimately inhibiting the Wnt/β-catenin signaling pathway [76] (Figure 3).

In stark contrast to the renal-specific validation of USP36, most USP members’ regulatory mechanisms on the Wnt/β-catenin pathway are derived exclusively from oncology and basic signaling research, necessitating cautious interpretation before clinical translation to kidney disease. Most USP members’ regulatory mechanisms on the Wnt/β-catenin pathway are derived from cancer and basic signaling pathway research. The mechanisms promoting Wnt signaling include USP2a, USP20, and USP4 enhancing β-catenin stability through deubiquitination [77,78]. Most importantly, extrapolating these oncology-derived targets reveals profound context-dependent contradictions that complicate their therapeutic potential. The most striking example is USP4, which exhibits a paradoxical, biphasic regulation of the Wnt pathway. On one hand, studies in colorectal cancer have found that USP4 stabilizes β-catenin through deubiquitination [79]. On the other hand, in a contradictory inhibitory loop, after activation, NLK causes USP4 to accumulate in the nucleus and target downstream transcription factor T cell factor 4 (TCF4) to inhibit its transcriptional activity, thereby inactivating the Wnt/β-catenin signaling pathway [80]. This contradictory dual role of USP4—acting simultaneously as an upstream activator and a downstream repressor of the same fibrotic pathway—highlights the extreme complexity of DUB signaling. The USP6 oncogene promotes the Wnt signaling pathway by deubiquitinating the Frizzled protein [81]. Additionally, USP14 may regulate Wnt signal transduction by deubiquitinating Dishevelled [82].

### 4.2. Inflammation

**Figure 4:**
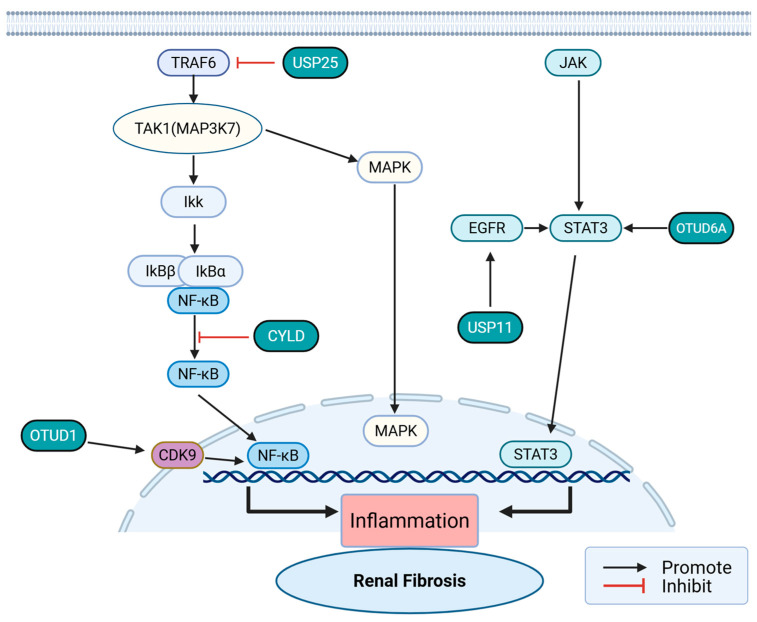
USPs regulate renal fibrosis through inflammatory response. USP25 inhibits the NF-κB signaling pathway by deubiquitinating TRAF6, and CyLD inhibits the NF-κB signaling pathway by reversing its K48-linked ubiquitination. OTUD1 promotes the phosphorylation of CDK9 through deubiquitination, thereby enhancing NF-κB activity. OTUD6A also promotes the phosphorylation and nuclear translocation of STAT3 through deubiquitination, thereby exacerbating angiotensin II (Ang II)-induced renal fibrosis.

Kidney inflammation is induced by various injuries (such as inflammation, diabetes, hypertension, ischemia–reperfusion, etc.), and persistent inflammation leads to the activation of pro-inflammatory cytokines, chemokines, and immune cells, driving fibrosis in acute and chronic kidney diseases [83]. The activation of NF-κB is one of the initiating events of kidney injury, driving the transcription of pro-inflammatory cytokines (IL-1β, IL-6, TNF-α) and adhesion molecules, thereby recruiting white blood cells into the kidney. The activity of NF-κB is strictly regulated by ubiquitination, particularly the K48-linked ubiquitination and degradation of IκBα (NF-κB inhibitor protein), as well as the K63-linked ubiquitination of upstream kinases such as TAK1 and tumor necrosis factor receptor-related factor 6 (TRAF6). The regulatory role of USP members in this process has been confirmed [84,85]. CyLD blocks the release of NF-κB by reversing ubiquitination of TRAF6 and K48 linkage, and effectively inhibits the NF-κB signaling pathway [86]. And in the diabetic nephropathy model, USP25 inhibits the overactivation of downstream NF-κB and MAPK pathways by deubiquitinating the E3 ligase TRAF6 [87]. However, in ischemic stroke and bacterial infection models, USP25 exhibits the opposite pro-inflammatory driving ability [88]. This stark phenotypic divergence suggests that USP25’s regulatory logic is acutely dependent on the specific pathogenic trigger or tissue microenvironment. Consequently, targeting USP25 systemically may yield unpredictable and potentially detrimental off-target effects, necessitating cautious, kidney-targeted delivery strategies if it is to be pursued clinically. OTUD1 promotes the phosphorylation of CDK9 through deubiquitination, enhancing NF-κB activity [89]. OTUD6A similarly promotes the phosphorylation and nuclear translocation of STAT3 through deubiquitination, increasing angiotensin II (Ang II)-induced kidney fibrosis [90]. In a Usp13^−/−^ mouse model study, it was found that USP13 inhibits the degradation of NLRP3 by trimming the K48 chain at K192 and K496 sites, thereby promoting the activation of the NLRP3 inflammasome at the initiation stage [91] (Figure 4).

In contrast to these established renal targets, the evidence for several other DUBs in kidney inflammation remains largely descriptive or extrapolated from in vitro or non-renal systems. Their specific in vivo contributions to renal fibrosis remain to be rigorously tested. USP7 utilizes its unique ubiquitin-like domain 2 (UBI2) to deubiquitinate the NF-κB subunit to prevent its degradation [92]. Meanwhile, it has also been found in endothelial biology research that USP7 stabilizes pyruvate dehydrogenase kinase 1 (PDK1) through deubiquitination, thereby promoting the activation of the Akt/NF-κB signaling axis, enhancing the activity of inflammatory pathways in endothelial cells [93].

### 4.3. Oxidative Stress

Oxidative stress occurs when the disruption of the redox system leads to a significant overproduction of reactive oxygen species (ROS), exceeding the capacity of the antioxidant system, which then triggers ferroptosis [94,95,96]. Oxidative stress and ferroptosis further impair mitochondrial function, resulting in insufficient energy production and additional ROS generation [97]. Additionally, this process exacerbates renal fibrosis by promoting inflammation and releasing pro-fibrotic factors. Backed by solid in vivo data, research indicates that USP10 promotes renal interstitial fibrosis. The study found that the expression levels of antioxidant genes Superoxide Dismutase 2 (SOD2) and ATP8 in the kidney tissue of UUO mice were significantly lower than those in the control group. That is, it exacerbates oxidative stress by ubiquitination and stabilizing the P53 protein [59]. In the diabetic nephropathy podocyte model, USP22 deubiquitinates and stabilizes long-chain acyl-CoA synthase (ACSL4). ACSL4 is a key executioner enzyme in ferroptosis, responsible for incorporating polyunsaturated fatty acids into phospholipids, making them susceptible to oxidation. Activation of the USP22-ACSL4 axis leads to the accumulation of lipid peroxides, inducing podocyte ferroptosis and loss [98]. USP13 is a deubiquitinase for *Beclin-1*, crucial for autophagosome formation. During the acute injury phase, USP13 deficiency leads to impaired autophagy, mitochondrial accumulation, and exacerbated cell death [99]. Although this conclusion was discovered in pulmonary fibrosis research, it was also found in an acute kidney injury model that renal tubular cells utilize the “secretory autophagy” mechanism to secrete fibroblast growth factor 2 (FGF2) extracellularly. As a potent paracrine factor, FGF2 activates interstitial fibroblasts. In this process, the autophagy stabilization mediated by USP13 may actually promote FGF2 release, thereby driving fibrosis progression [100]. Conversely, providing a protective target, USP9X plays a protective role in diabetic renal fibrosis by activating the Nrf2-ARE antioxidant pathway, reducing the upregulation of FN and TGF-β1 induced by advanced glycation end products (AGEs), and lowering the excessive production of ROS [101].

In addition, Nrf2 is the master switch for cellular antioxidant responses, while Keap1 is its cytosolic inhibitor. In fibrosis research, it was found that USP7, through ubiquitin removal of Keap1, prevents cells from effectively initiating the transcription of antioxidant genes [102]. In digestive system research, under oxidative stress (H_2_O_2_ stimulation) conditions, USP7 ubiquitin removes and stabilizes the autophagy regulatory protein AMBRA1, causing it to competitively bind with Nrf2, interfering with the stabilizing effect of the protective deubiquitinase DUB3 on Nrf2, and indirectly promoting Nrf2 degradation [103,104]. During the process of converting ischemia–reperfusion injury (IRI) to fibrosis, USP7-stabilized DNMT1 enters the nucleus, silences the gene promoter *FMR1*, and promotes the occurrence of ferroptosis through an unknown mechanism [105].

### 4.4. Cell Cycle

**Figure 5:**
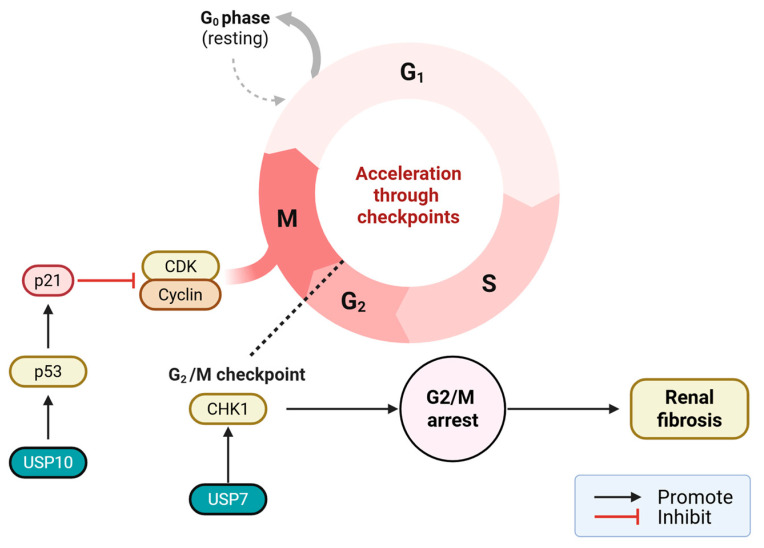
USPs regulate renal fibrosis through cell cycle arrest and senescence. USP10 stabilizes P53 activation, leading to the downstream target gene p21 and arresting RTECs in the G2/M phase. USP11 stabilizes EGFR, inducing cell cycle arrest in the G2/M phase. USP7 stabilizes CHK1, causing cell cycle arrest in the G2/M phase.

While transient cell cycle arrest serves as a critical protective mechanism to prevent the replication of damaged DNA, prolonged G2/M phase arrest of renal tubular epithelial cells (RTECs) is closely associated with the progression of renal fibrosis, creating a temporal paradox that complicates therapeutic intervention. When RTECs attempt to repair after severe damage, if the DNA damage is too severe or stress signals persist, the cells will arrest in the G2/M phase. These arrested cells do not die and do not complete division; instead, they enter a state similar to senescence, known as the Senescence-Associated Secretory Phenotype (SASP). G2/M arrest drives fibrosis because this specific cell cycle state activates downstream signaling cascades such as the JNK pathway and the Hypoxia-Inducible Factor 1-α (HIF-1α)/p53 axis [106,107]. USP10 specifically removes the K48 ubiquitin chain from the p53 protein, preventing its degradation. Stable p53 enters the nucleus and activates the downstream target gene p21, leading to its sustained high expression and causing RTECs to arrest in the G2/M phase [59]. USP11 deubiquitinates extranuclear epidermal growth factor receptor (EGFR), promoting its stability. Sustained EGFR signaling drives partial EMT, inducing cell cycle arrest at the G2/M phase [108,109]. Research has found that the ubiquitin-like protein FAT10 can directly bind and stabilize USP7, while USP7 subsequently deubiquitinates and stabilizes CHK1. Persistent high expression of CHK1 leads to prolonged cell cycle arrest in the G2/M phase [110]. In addition, studies on fibroblasts have found that USP7 is a novel target for senescence cell clearance [111] (Figure 5).

### 4.5. Others

In the kidney IRI model, Li et al. found that USP14 expression was significantly increased. The study confirmed that USP14 does not directly act on mitochondrial proteins but deubiquitinates and stabilizes the transcription factor Tfap2α (transcription factor AP-2α). The stabilized Tfap2α enters the nucleus and inhibits the transcription of TANK-binding kinase 1 (TBK1). TBK1 is a key kinase for the phosphorylation of mitochondrial autophagy receptors. The activation of the USP14-Tfap2α axis leads to a decrease in TBK1 levels, thereby blocking the recognition and engulfment of damaged mitochondria by autophagosomes. As a result, damaged mitochondria accumulate, apoptosis increases, and fibrosis worsens [112,113]. In damaged mitochondria, *PINK1* recruits the E3 ligase Parkin, which coats the mitochondrial surface with ubiquitin chains. USP30 acts as a “negative regulator” (the brake) of this process by removing those ubiquitin chains [114,115]. Recent research has found that USP11 deubiquitinates the transcription factor Krüppel-like factor 4 (KLF4), thereby inducing pyroptosis in renal tubular cells and releasing inflammatory factors that exacerbate fibrosis [116]. In the hyperglycemic environment of diabetic nephropathy (DKD), USP22 expression is upregulated. It stabilizes Snail1, a key transcription factor in EMT, by deubiquitinizing Snail1, leading to E-cadherin loss and fibrosis [117].

## 5. Anti-Fibrotic Therapies Related to USP

The development of USP-related drugs is usually carried out through different pathophysiological mechanisms regulated by different members of the USP family. Vialinin A, a USP4 inhibitor, has been proven to alleviate liver fibrosis in the liver through the mTOR signaling pathway. USP4 is a key stabilizing factor for TβRI in the TGF-β pathway and holds the same potential in kidney fibrosis [52,118,119]. Mitoxantrone was originally a chemotherapeutic drug used to treat multiple sclerosis and prostate cancer and was identified as a potent USP11 inhibitor. Its pharmacological knockdown promoted the degradation of EGFR and weakened the activation of TGF-β1 and its downstream signaling pathways [108]. In a mouse kidney fibrosis model induced by UUO, administration of USP2 inhibitor, ML364, can block this process. By inhibiting USP2, ML364 disrupts the stability of the TGF-β receptor and reduces the expression of Collagen I and α-SMA in kidney tissue, thereby significantly alleviating interstitial fibrosis [55]. Spautin-1 administered to UUO model mice significantly inhibited USP10, reversed the cell cycle arrest through p53, and subsequently reduced the expression of fibronectin and inflammatory cell infiltration, showing the potential value of Spautin-1 in CKD treatment [59]. bAP-15 is a specific inhibitor of p300 protein stability mediated by deubiquitination in human renal tubular epithelial cells, and USP14 and UCHL5 jointly stabilize the histone acetyltransferase p300. The specific inhibitor bAP-15 inhibits the transcription of profibrotic genes while also suppressing the EMT process mediated by p300 [120]. Quercetin has been proven to significantly inhibit the expression and activity of USP22, disrupt the USP22-Snail1 interaction and promote Snail1 degradation, thereby effectively reversing diabetic tubulointerstitial fibrosis in in vivo and in vitro models [121]. USP21 deubiquitinates and stabilizes HIF-1α. Kidney fibrosis is accompanied by severe tissue hypoxia, and the sustained activation of HIF-1α, while potentially protecting cells in the early stages, promotes fibrosis gene expression in the chronic stage. The potential of the novel USP21 inhibitor Baicalein in anti-kidney fibrosis is being explored [122]. In small cell lung cancer, HBX41,108 inhibits USP7 [123]. PR-619 is a non-selective broad-spectrum DUB inhibitor. In the UUO model, PR-619 specifically reduced the protein level of SMAD4, demonstrating anti-fibrotic effects, but the specific intermediate mechanisms of the specific members involved have not been elucidated. Considering its relevance to SMAD4 stability, such as USP9x or USP10, it has the potential to act as a USP regulator to modulate fibrosis [124] (Table 1). When prioritizing the most robustly validated targets for clinical translation, USP11 and USP30 currently emerge as the leading candidates. USP11 has demonstrated strong in vivo efficacy across multiple fibrotic models, supported by the availability of the potent, albeit systemically toxic, inhibitor Mitoxantrone [108]. Even more promisingly, USP30 represents the most translationally advanced target, with the highly selective inhibitor MTX652 recently entering Phase II clinical trials for acute kidney injury [115].

Despite these promising preclinical findings, the clinical translation of USP inhibitors for renal fibrosis faces substantial challenges, primarily concerning selectivity and off-target effects. Because the catalytic domains of USPs are highly conserved, achieving high selectivity is difficult, and broad inhibition can disrupt essential cellular homeostasis [125]. For instance, mitoxantrone is a well-known topoisomerase II poison that induces severe, dose-dependent cardiotoxicity [126,127]. Furthermore, translational feasibility is currently limited by a lack of kidney-targeted delivery systems and poor in vivo pharmacokinetic profiles [128]. Overcoming these limitations in drug development requires the design of next-generation allosteric inhibitors, targeted protein degraders (such as PROTACs), and the advancement of highly selective agents, etc., to ensure strict renal safety and efficacy.

## 6. Discussion and Future Perspectives

As our understanding of ubiquitin signaling in renal pathology deepens, research on the USP family has reached a watershed moment, transitioning from mechanistic elucidation to the frontier of clinical translation. A major conceptual advance in recent years is the paradigm shift from viewing the ubiquitin–proteasome system solely as a cellular degradation machinery to recognizing USPs as highly precise, dynamic regulators of profibrotic signaling cascades, such as the TGF-β/SMAD, inflammatory, and cell death pathways. However, current knowledge remains fragmented regarding the complexity of the ubiquitin proteome. Critical unresolved questions remain regarding the highly context-dependent substrate networks of individual USPs, as Transforming Growth Factor-beta 1 (TGF-β1) stands as the preeminent pro-fibrotic cytokine driving renal fibrosis among the three TGF-β isoforms in mammals their functions can vary drastically across different renal cell types and disease stages. A major unmet challenge is deciphering how to restore the delicate ubiquitination/deubiquitination homeostasis rather than pursuing a blunt strategy of complete inhibition. At the same time, future studies must delineate the spatiotemporal specificity of USPs—distinguishing their roles during the distinct phases of injury (e.g., the AKI-to-CKD transition) versus established fibrosis. Furthermore, the clinical translation of broad-spectrum USP inhibitors is currently hindered by severe systemic off-target toxicities and a lack of renal specificity. To circumvent the off-target toxicity that has plagued previous antifibrotic agents, the next generation of therapeutics should prioritize precision modulation. Future research may leverage cutting-edge technologies, such as Artificial Intelligence (AI)-driven drug discovery and Proteolysis Targeting Chimeras (PROTACs), to achieve highly selective regulation of specific profibrotic USPs. Additionally, integrating these selective agents, alongside allosteric regulators, with kidney-targeted nanocarriers will be meaningful to maximize renoprotection, minimize systemic side effects, and may bridge the gap between bench research and clinical application.

## 7. Conclusions

In conclusion, this review offers a thorough overview of the structural foundations and biological origins of USPs. It systematically outlines their specific molecular mechanisms and regulatory functions in the progression of renal fibrosis. Furthermore, by analyzing the development progress of existing medicines, we emphasized the potential of anti-fibrotic therapies related to USPs as a promising treatment strategy. Although the development of pharmacological USP inhibitors faces initial challenges, their significant impact on the progression of kidney disease presents a hopeful therapeutic opportunity. Understanding the specific mechanism networks of USPs and combining this knowledge with next-generation precision medicine design may be crucial for realizing their potential as effective anti-fibrotic treatments.

## Figures and Tables

**Table 1:** USP is involved in the promotion and inhibition of renal fibrosis.

UPS	Substrates	Effect on Fibrosis	Mechanism	Tool Medicine	Ref.
USP2	TXNDC5	Promotive	Promotes TGF-β/SMAD signaling by stabilizing TβRI	ML364	[55]
USP4	TβRI	Promotive	Promotes TGF-β/SMAD signaling by stabilizing TβRI	Vialinin A	[52]
USP7	CHK1	Promotive	Promotes cell cycle arrest by stabilizing CHK1 Inhibits Wnt/β-catenin signaling by stabilizing Axin	HBX41,108	[110]
USP9X	Nrf2	Inhibitory	Inhibits oxidative stress by activating the Nrf2-ARE antioxidant pathway	PR-619	[101]
USP10	p53	Promotive	Promotes TGF-β/SMAD signaling and inhibiting oxidative stress by stabilizing p53, promotes cell cycle arrest	Spautin-1, PR-619	[59,60,61]
USP11	TβRI TβRII EGFR KLF4	Promotive	Promotes TGF-β/SMAD signaling by stabilizing TβRI Promotes TGF-β/SMAD signaling by stabilizing TβR II Promotes cell cycle arrest Induces pyroptosis in renal tubular cells and releases inflammatory factors that exacerbate fibrosis by stabilizing KLF4	Mitoxantrone	[53,54,108,109,116]
USP14	Tfap2a	Promotive	Blocks autophagosome recognition and phagocytosing damaged mitochondria promotes fibrosis	-	[112,113]
USP22	ACSL4 Snail1	Promotive	Inhibits oxidative stress by stabilizing ACSL4 Leading to E-cadherin loss and fibrosis by stabilizing Snail1	Quercetin	[98]
USP25	SMAD4 TRAF6	Inhibitory	Inhibits TGF-β/SMAD signaling by altering the activity and function of SMAD4 Inhibits NF-κB signaling and MAPK signaling by altering the activity and function of TRAF6	-	[63,87]
USP30	TOM20/Miro1	Inhibitory	Promotes the clearance of dysfunctional mitochondria through PINK1/Parkin signaling pathway	MTX652	[114,115]
USP36	DOCK4	Promotive	Promotes Wnt/β-catenin signaling by stabilizing DOCK4	-	[75]
USP38	STRAP	Promotive	Promotes TGF-β/SMAD signaling by promoting SMAD2/3 phosphorylation and nuclear translocation	-	[62]
CYLD	IκBα	Inhibitory	Inhibits NF-κB signaling and inflammation by stabilizing IκBα	-	[86]
OTUD1	CDK9	Promotive	Promotes NF-κB signaling and inflammation by facilitating the phosphorylation of CDK9	-	[89]
OTUD6A	STAT3	Promotive	Promotes STAT3 phosphorylation and nuclear translocation	-	[90]

## Data Availability

No data was used for the research described in the article.

## Abbreviations

The following abbreviations are used in this manuscript:

USP	Ubiquitin-specific protease
DUB	Deubiquitinating enzyme
PTM	Post-translational modification
CKD	Chronic kidney disease
HGNC	Human Genome Nomenclature Committee
UBP	Ubiquitin-Specific Processing Protease
UBD	Ubiquitin-binding domain
ZnF-UBP	Zinc-finger ubiquitin binding protein
DUSP	USP-specific protease domains
UBL	Ubiquitin-like domains
DNMT1	DNA methyltransferase 1
WDR	WD40-repeats
LAP	Latent-related peptide
LTBP	latent TGF-β-binding protein
TGF-β1	Transforming Growth Factor- β 1
ECM	extracellular matrix
TβRI	TGF-β type I receptor
TβRII	TGF-β type II receptor
STRAP	Serine-Threonine Kinase Receptor-Associated Protein
GEF	Guanine nucleotide exchange factor
EMT	Epithelial-to-mesenchymal transition
TCF4	Transcription factor T cell factor 4
TRAF6	Tumor necrosis factor receptor-related factor 6
UBI2	Unique ubiquitin-like domain 2
IκBα	NF-κB inhibitor protein
Ang II	Angiotensin II
NLRP3	NOD-, LRR- and pyrin domain-containing protein 3
ROS	Reactive oxygen species
SOD2	Superoxide Dismutase 2
ACSL4	Long-chain acyl-CoA synthase
UBI2	Ubiquitin-like domain 2
PDK1	Pyruvate dehydrogenase kinase 1
FN	Fibronectin
α-SMA	α-smooth muscle actin
AGEs	Advanced glycation end products
FGF2	Fibroblast growth factor 2
IRI	Ischemia–reperfusion injury
RTECs	Renal tubular epithelial cells
HIF-1α	Hypoxia-Inducible Factor 1-α
EGFR	Epidermal Growth Factor Receptor
SASP	Senescence-Associated Secretory Phenotype
Tfap2α	Transcription factor AP-2α
TBK1	TANK-binding kinase 1
KLF4	Krüppel-like factor 4
DKD	Diabetic nephropathy
UUO	Unilateral ureteral obstruction
PROTACs	Proteolysis Targeting Chimeras
TXNDC5	thioredoxin domain containing 5
